# Multidisciplinary Oncology Education Among Postgraduate Trainees: Systematic Review

**DOI:** 10.2196/63655

**Published:** 2025-05-26

**Authors:** Houman Tahmasebi, Gary Ko, Christine M Lam, Idil Bilgen, Zachary Freeman, Rhea Varghese, Emma Reel, Marina Englesakis, Tulin D Cil

**Affiliations:** 1 Temerty Faculty of Medicine, University of Toronto, Toronto, ON, Canada; 2 Department of Surgery, University of Toronto, Toronto, ON, Canada; 3 School of Medicine, Koç University, Istanbul, Turkey; 4 Faculty of Science, Wilfrid Laurier University, Waterloo, ON, Canada; 5 Sprott Department of Surgery, Princess Margaret Cancer Centre, University Health Network, 6th floor, 700 University Ave, Toronto, ON, M5G 1X6, Canada, 1 416 946 4501 ext 3984, 1 416 946 4429; 6 Library and Information Services, University Health Network, Toronto, ON, Canada

**Keywords:** multidisciplinary, oncology, postgraduate medical education, resident, fellow, surgery, hematology, radiation oncology, geriatrics, palliative

## Abstract

**Background:**

Understanding the roles and patient management approaches of the entire oncology team is imperative for effective communication and optimal cancer treatment. Currently, there is no standard residency or fellowship curriculum to ensure the delivery of fundamental knowledge and skills associated with oncology specialties with which trainees often collaborate.

**Objective:**

This study is a systematic review that aims to evaluate the multidisciplinary oncology education in postgraduate medical training.

**Methods:**

A systematic literature search was performed using MEDLINE, Embase, Cochrane Database of Systematic Reviews, Cochrane CENTRAL, APA PsycINFO, and Education Resources Information Center in July 2021. Updates were performed in February 2023 and October 2024. Original studies reporting the effectiveness of multidisciplinary oncology training among residents and fellows were included.

**Results:**

A total of 6991 studies were screened and 24 were included. Fifteen studies analyzed gaps in existing multidisciplinary training of residents and fellows from numerous fields, including surgical, medical, and radiation oncology; geriatrics; palliative medicine; radiology; and pathology programs. Trainees reported limited teaching and knowledge of oncology outside of their respective fields and endorsed the need for further multidisciplinary oncology training. The remaining 9 studies assessed the effectiveness of educational interventions, including tumor boards, didactic sessions, clinical rotations, and case-based learning. Trainees reported significant improvements in multidisciplinary oncology knowledge and skills following the interventions.

**Conclusions:**

These data suggest postgraduate medical trainees have limited formal multidisciplinary oncology training. Existing educational interventions show promising results in improving trainees’ oncology knowledge and skills. There is a need for further research and the development of multidisciplinary oncology curricula for postgraduate medical training programs.

## Introduction

Cancer was the second leading cause of death in the United States in 2023 [1]. Cancer care often requires a team of physicians including surgical, medical, and radiation oncologists, as well as specialists in radiology and pathology [2]. Knowledge of collaborating oncologists’ roles and appropriate multidisciplinary referrals may impact cancer treatment. There is evidence of improved adherence to standard treatment guidelines with multidisciplinary referrals for patients with prostate [3], lung cancer [4], and bladder cancer [5].

There is considerable potential to improve interdisciplinary communication between various oncologic specialists and to optimize psychosocial support for patient care. Therapies with different oncologists must be well coordinated and specifically selected based on the medical and social needs of each patient. To achieve this, knowledge of other disciplines’ roles, responsibilities, and treatment options is necessary for effective communication and optimal cancer care.

There is currently no standard curriculum for delivering multidisciplinary oncology education in residency and fellowship programs in the United States [6-10]. Mattes et al [11] identified that while many of the program requirements for oncology subspecialties emphasize the importance of providing multidisciplinary cancer care, how this occurs varies widely between subspecialties. Not all programs mandate multidisciplinary oncology rotations or experiential specialty training, and only a select few require attendance at multidisciplinary tumor board meetings (MTBM) [6-12]. Such a training gap may impact trainee education and, as a result, influence referral patterns and the timely access of patients to multimodal cancer therapies.

The objective of this study was to perform a systematic review of the literature to evaluate the multidisciplinary oncology education in postgraduate medical training (ie, interns, residents, and fellows). This study provides a review of literature analyzing the education of learners about the role of any collaborating physician specialty involved in oncology care, including but not limited to, medical oncology, radiation oncology, surgical oncology, and palliative care. These data summarize gaps in training programs identified across studies, the suggested educational interventions to bridge these gaps, and limitations in the literature within the field.

## Methods

### Research Design and Methodology

This systematic review was reported based on PRISMA (Preferred Reporting Items for Systematic Reviews and Meta-Analyses) guidelines [13]. The protocol was registered and published by PROSPERO (ID: CRD42022271308).

### Search Strategy

A search strategy was developed with the assistance of an information specialist using these and other related terms: “Residents or Fellows or Trainees or Medical Training” AND “Education or Training Programs” AND “Multidisciplinary” AND “Oncology.” The following databases were searched from inception: MEDLINE, MEDLINE In-Process, Embase Classic + Embase, Cochrane Central Register of Controlled Trials, Cochrane Database of Systematic Reviews, and APA PsycINFO (all via the Ovid platform); and Education Resources Information Center via the EbscoHost platform. The search was initially performed on July 21, 2021, and updated twice (ie, on February 26, 2023, and October 9, 2024). Table S1 in Multimedia Appendix 1 shows the number of citations identified from each database. The search strategy and the number of citations identified via MEDLINE are included in Table S2 in Multimedia Appendix 1.

### Eligibility Criteria

Eligibility criteria were developed prior to the search strategy. The scope of this study was to evaluate the multidisciplinary oncology education offered by residency and fellowship programs to postgraduate medical trainees. Thus, the first eligibility criterion was the inclusion of studies investigating postgraduate medical training (ie, interns, residents, and fellows). Studies about nonphysician specialties (eg, nursing, pharmacy, or dentistry), attending or staff physicians, or those involving solely Masters, PhD, or medical students were excluded. Studies were included if their focus was specific to oncology care. Selected studies focused on multidisciplinary aspects of medical education, which included knowledge of collaborating medical specialties and their roles in cancer care (eg, surgical trainees’ knowledge of radiation or medical treatments). Trainees from all specialties were included, as long as the study was assessing the multidisciplinary oncology education of trainees, and therefore, these were not necessarily restricted to oncology residency or fellowship programs (eg, medical oncology, radiation oncology, surgical oncology). Only primary research papers and studies available in English (ie, both original and translations to English) were included. Thus, all reviews, case studies, opinion papers, abstract-only papers, conference literature, and short reports were excluded.

### Study Selection

There were 2 stages of review: title and abstract screening, followed by full-text screening. A total of 6 reviewers (HT, GK, CML, IB, ZF, and RV) were involved, and studies were screened by a minimum of 2 independent reviewers at each stage. Discrepancies were resolved by a third reviewer. Both screening stages were performed on Covidence [14], a web-based systematic review organization software.

### Data Extraction and Synthesis

Data extraction was performed on the selected studies. Studies were divided between 3 reviewers (HT, CML, and RV) who performed data extraction. Study design, study population, outcome measures, and main results were extracted from each study.

### Quality Assessment

Selected studies were independently assessed for quality by 2 independent reviewers (CL, IB, and RV) using the Mixed Methods Appraisal Tool (MMAT) version 2018 [15]. Discrepancies were resolved through discussion with a third author (HT). The MMAT was chosen due to its ability to concomitantly assess multiple study types (ie, qualitative, quantitative randomized controlled trial, quantitative nonrandomized, quantitative descriptive, or mixed methods). Each study was evaluated on a set of 5 criteria depending on the study type. For survey studies, the risk of nonresponse bias was deemed to be high if the response rate was below 70%. Studies were assigned an overall quality score ranging from 0 to 5 stars based on the number of criteria that were met.

## Results

### Study Characteristics

**Figure 1.**
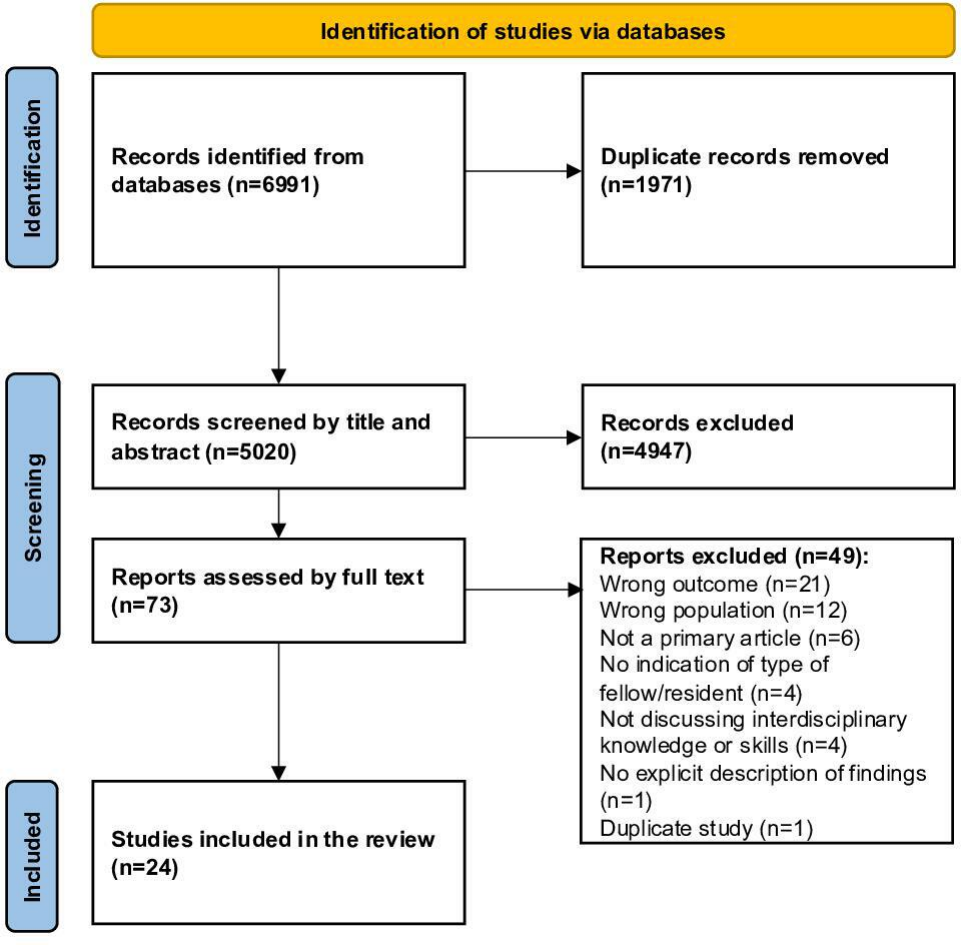
PRISMA (Preferred Reporting Items for Systematic Reviews and Meta-Analyses) diagram of the systematic review. Adapted from Page et al [13].

The search strategy resulted in a total of 6991 studies. After removing duplicates between databases, 5020 unique studies were identified. A total of 73 studies remained after title and abstract screening. Full-text screening excluded 49 studies, and 24 studies were therefore included in the final analysis. The PRISMA flow diagram is demonstrated in Figure 1.

The remaining 24 studies were divided into 2 categories. Fifteen studies assessed the quality of existing postgraduate oncology training based on trainees’ multidisciplinary knowledge. The remaining 9 assessed trainees’ multidisciplinary knowledge following an educational intervention. For the latter category, all studies with educational interventions directed toward improving multidisciplinary oncology knowledge and skills among interns, residents, and fellows were included. These included studies that are part of the formal postgraduate medical training (eg, residency or fellowship program), as well as external initiatives for improving multidisciplinary oncology training. Studies involving educational interventions for medical students and staff or attending physicians were not included.

### Existing Multidisciplinary Training

A summary of the 15 studies evaluating the impact of existing multidisciplinary oncology training is included in Table 1. These studies included surgical or surgical oncology fields [16-23], hematology or medical or hematology oncology [16,20-22,24,25], geriatrics or geriatric oncology [20,22,26], radiation oncology [16,20,21,23,27], palliative medicine [16,20], radiology [21], pathology [21], genetics [23], dermatology [23], pediatric specialties [28], and other medical fields (eg, internal medicine, nephrology, neurology) [22,23].

**Table 1.** Summary of studies evaluating the existing multidisciplinary education across postgraduate medical training programs.

Reference	Study design	Sample	Outcome measure	Main findings and conclusions
Akthar et al[16]	Electronic surveys completed by oncology trainees and program directors across the United States in 2013	557 hematology or medical oncology, surgical oncology, radiation oncology, and palliative medicine residents and fellows 141 hematology or medical oncology, surgical oncology, radiation oncology, and palliative medicine program directors	Proportion of trainees who received formal education in oncology fields outside of their specialty	Generally limited interdisciplinary oncology education: ≤70% of trainees reported receiving formal interdisciplinary education; highest rate of training in radiation oncology (70% of trainees) and lowest rate of training in geriatric oncology (19% of trainees) Consistently lower rates of interdisciplinary oncology training reported by trainees compared to program directors (*P*<.01)
Brenner and De Donno[17]	Survey of postgraduate year 1‐5 residents from 3 general surgery programs: Florida Atlantic University, The University of Iowa, and The University of Connecticut	135 general surgery residents	Proportion of residents who indicated receiving training in a specific multidisciplinary field	Limited proportion of residents indicated receiving multidisciplinary training: radiation oncology (23%), chemotherapy (31%), and palliative medicine (53%) Majority (82%) of residents endorsed further multidisciplinary training
David et al[25]	Survey of hematology residents (ie, postgraduate years 4‐5) or fellows (ie, postgraduate year 6‐7) across Canada as part of a cross-sectional study	29 hematology residents and 3 hematology fellows	Geriatric oncology curriculum needs assessment	56.3% did not receive geriatric oncology teaching 96.9% endorsed the inclusion of geriatric training in hematology residency
Delaye et al[22]	Surveys completed by French residents and senior physicians regarding the field of onco-nephrology	Residents (n=130) and senior physicians (n=98) from nephrology, oncology, hematology, surgery and geriatrics	Current practices in onco-nephrology, information resources, existing cooperation networks, and expectations about onco-nephrology	Oncology residents rated their confidence in facing renal events as 5.5/10 Nephrology residents rated their confidence in facing cancer events as 6.0/10 21% of residents had received onco-nephrology teaching, which was judged as insufficient
Eid et al[24]	Review of literature, expert consultation, review of fellows’ rotation evaluations, and interviews with current and recently graduated fellows, as a means of needs assessment for the development of a geriatric oncology program at MD Anderson Cancer Center	9 current hematology-oncology fellows (years 1‐3) at MD Anderson Cancer Center 2 MD Anderson faculty members who recently graduated from a hematology-oncology fellowship	Geriatric oncology training program needs assessment	Top 3 identified needs for geriatric oncology programs, based on current educational gaps: Geriatric assessment Pharmacology knowledge Psychosocial knowledge
Givi et al[29]	Semistructured interviews with program directors and faculty in head and neck surgery across the United States and Canada over a 7-month period	58 participants including head and neck surgery program directors and faculty	Head and neck surgical oncology training needs assessment	38% endorsed increasing the number of head and neck surgery fellows’ interactions with medical oncology, radiation oncology, and speech and language pathology 85% view exposure to multidisciplinary teams as essential in training curricula
Le Nail and Samargandi[30]	Web-based questionnaire on various aspects of MTBMs^a^ completed by French orthopedic oncology residents	27 orthopedic oncology residents	Residents’ opinions on educational impact and areas of improvement for MTBMs	54% agreed that MTBM is an appropriate venue for teaching 75% endorsed that MTBMs improved their knowledge of other specialties involved 71% indicated opportunities to improve teaching during MTBM, the most popular suggestion being active participation of residents (voted by 46% of all residents)
Maggiore et al[26]	Web-based survey completed by program directors of geriatrics fellowship programs in the United States	67 geriatrics program directors	Proportion of program directors offering or endorsing future learning opportunities in the field of geriatric oncology	Majority (81%) of program directors offered didactic teaching in the form of formal geriatric oncology lectures/seminars Limited number of program directors offered clinical experience: 39% offered mandatory oncology clinical experience and 46% offered clinical electives Majority (77%) endorsed oncology training as part of the geriatrics fellowship
Mäurer et al[23]	Web-based survey distributed to all junior oncology groups represented in Young Oncologists United in Germany regarding interdisciplinarity in oncology	294 participants including 268 physicians (staff and trainees) from internal medicine, gynecology, radiotherapy and radiation oncology, general surgery, genetics, neurosurgery, urology, neurology, and dermatology	Opinions on interdisciplinarity at clinic, educational, and research levels	63.1% assigned a high priority to interdisciplinary residency training Only 18.3% had the opportunity to participate in rotations in other specialties beyond their curriculum 71.4% were interested in participating in rotations in other specialties 73.1% of those who completed interdisciplinary rotations benefited from them
Morris et al[27]	Web-based survey completed by radiation oncology residents in Australia, New Zealand, and Singapore	61 radiation oncology residents	Proportion of residents who indicated receiving or endorsing future geriatric oncology training	Majority (91.8%) did not receive any geriatric training Limited number of residents (39.3%) comfortable managing complex geriatric issues Majority (85.3%) endorsed additional geriatric training
Morris et al[20]	2-stage Delphi consensus with input from a panel of internationally recognized oncology experts, staff physicians, radiation oncology and clinical oncology trainees, allied health professionals, patients, and caregivers. Experts were from geriatrics, geriatric oncology, and radiation oncology. Staff physicians were from clinical/medical oncology, palliative care, and surgical oncology.	A total of 103 and 54 individuals participated in rounds 1 and 2 of the modified Delphi consensus process, respectively Majority were radiation oncologists (43%)	Establishing learning outcomes for a geriatric radiation oncology curriculum	33 learning outcomes identified in the areas of fundamental geriatric medicine concepts, epidemiology, geriatric screening, planning and delivery of radiation therapy, geriatric palliative care, surgery, systematic treatment, research, communication skills, and health advocacy
Park et al[18]	30 item self-efficacy survey completed by residents at Ohio State University Wexner Medical Center, in order to measure knowledge and skills in 6 breast cancer care aspects: genetics, surgery, medical oncology, radiation oncology, pathology, and radiology	31 general surgery residents	Residents’ perceived capability (ie, self-efficacy score) in various domains of breast cancer care	Highest self-efficacy in surgery (3.56/5) vs lowest in genetics (2.67/5), radiation oncology (2.67/5), and pathology (2.67/5) Significant improvement of self-efficacy in surgery only (*P*=.002) with additional years in residency
Picca and Reed[28]	Semistructured interviews with faculty and trainees across pediatric oncology, radiology, pathology, surgical oncology, and palliative care	4 pediatric oncology fellows 11 pediatric oncology, pathology, radiology, palliative care, and surgical oncology faculty physicians	Exploration of learning in tumor boards	Trainees found tumor board presentation to be educational Barriers to learning: competing clinical/administrative responsibilities Facilitators to learning: learning-focused goals, faculty mentorship during presentation preparation, collaborative discussion, content tailored to learners and board exams, and supportive environment Web-based tumor boards promoted accessibility and convenience but decreased learning due to limited engagement, discussion, and professional relationship development
Walraven et al[21]	Semistructured interviews with Dutch residents and specialists in medical/surgical/radiation oncology, radiology, nuclear radiology, and pathology participating in MDTMs^b^	19 residents 16 specialists	Residents’ barriers and facilitators to participate in MDTMs	100% agreed that MDTMs play an important role in both education and patient care Barriers: insufficient supervisor guidance, time constraints, meeting atmosphere and hierarchy, strict regulations, unfamiliarity, and resident’s personal characteristics Solutions: MDTM simulation training, and training courses on communication and meeting skills
Wilson et al[19]	Survey of applicants to Roswell Park Cancer Institute surgical oncology fellowship program	29 general surgery residents or recent general surgery graduates applying to surgical oncology fellowship	Proportion of applicants with breast surgery exposure and their comfort with medical and surgical management of breast cancer	Majority (65%) had exposure to multidisciplinary breast cancer clinics, involving medical and surgical oncologists Lower level of comfort (7.07/10) with breast cancer medical management compared to surgical management (7.34‐9.10/10 depending on the type of surgery)

a MTBM: multidisciplinary tumor board meeting.

b MDTM: multidisciplinary team meetings.

Thirteen studies obtained opinions of trainees with respect to multidisciplinary oncology education within their training programs [16-25,27,28,30]. Morris et al [20] used a Delphi consensus process, and 4 studies directly interviewed trainees and faculty [21,24,28,29]. The remainder of the studies used surveys. Maggiore et al [26] surveyed geriatrics program directors, Givi et al [29] surveyed head and neck surgery program directors, and Akthar et al [16] surveyed program directors of pediatric and adult hematology oncology, surgical oncology, radiation oncology, and palliative medicine. Eid et al [24] used a combination of expert consultation, trainee interviews, review of trainee rotation evaluations, and literature review to assess their multidisciplinary educational needs.

While all studies analyzed the quality of existing multidisciplinary education, there were differences in the disciplines investigated across studies. Akthar et al [16], Delaye et al [22]*,* Mäurer et al [23]*,* Walraven et al [21], Picca and Reed [28], and Brenner and De Donno [17] focused on identifying broad gaps in multidisciplinary education including knowledge and skills of trainees in numerous fields, such as radiation, surgical, and medical oncology, radiology, pathology, geriatrics, palliative medicine, and other pediatric and medical fields. The remaining 8 studies focused on a more specific set of trainee skills. David et al [25], Eid et al [24], and Maggiore et al [26] assessed gaps in geriatric oncology education among hematology residents and fellows, hematology oncology fellows, and geriatrics fellows, respectively. Morris et al [20,27] assessed gaps in the radiation oncology training curriculum. Park et al [18] and Wilson et al [19] assessed the quality of general surgery residency training in breast cancer care. Le Nail and Samargandi [30] evaluated the quality of tumor boards for orthopedic oncology trainees. Finally, Givi et al [29] performed a needs assessment analysis of the head and neck surgery training curriculum.

13 studies assessed the strengths and weaknesses of oncology training programs [16-19,21-23,25-30]. Of these, 11 found that trainees had limited exposure to multidisciplinary oncology disciplines, barriers to attending multidisciplinary oncology meetings, and a low level of trainee comfort in multidisciplinary oncology knowledge [16-19,21-23,25-28]. Givi et al [29] found that 27% of interviewees indicated exposure to multidisciplinary care as a strength of the head and neck surgery training program, although 38% endorsed the need to improve fellows’ multidisciplinary participation. In general, Akthar et al [16] found the least amount of multidisciplinary training in geriatric oncology, compared to palliative medicine, medical, radiation, and surgical oncology. Similarly, Morris et al [27] found that less than 10% of radiation oncology trainees received geriatrics training. Furthermore, less than half of geriatrics fellows were offered geriatric oncology rotations [26]. For multidisciplinary breast cancer management, Park et al [18] found limited training in genetics, radiation oncology, and pathology among surgical residents, compared to rotations within surgery, radiology, and medical oncology. Brenner and De Donno [17] found that a small proportion of general surgery residents received training in the fields of radiation (23%) and medical oncology (31%), but over half (53%) received exposure to palliative care.

Additionally, 11 studies researched areas of improvement for multidisciplinary oncology education among the postgraduate programs via surveys, interviews, Delphi consensus, and literature search [17,20,21,23-30]. Maggiore et al [26] and Morris et al [27] found that 77% of geriatrics fellows and 85.3% of radiation oncology residents advocated for further geriatric oncology training. David et al [25] found that over 95% of hematology trainees endorsed geriatric training during residency. 82% of general surgery residents surveyed by Brenner and De Donno [17] agreed that additional multidisciplinary training is needed to optimize cancer care. Additionally, based on an educational needs assessment, Eid et al [24] found that the top 3 priorities for a geriatric oncology program included geriatric assessment, pharmacology, and psychosocial skills. MTBMs were found to enhance trainee experience and multidisciplinary oncology education [21,28,30]. However, some barriers to attending meetings included time constraints, clinical duties, and lack of active resident participation [21,28,30]. Residents and specialists interviewed by Walraven et al [21] suggested that the educational value of multidisciplinary team meetings could be improved through additional training such as multidisciplinary team meeting simulations and courses on effective communication and meeting skills.

### Impact of Educational Interventions

A summary of the 9 studies analyzing the impact of educational interventions is included in Table 2. The majority included general surgery trainees [31-35]. Faculty and trainees from radiation oncology [32,35], medical oncology [12,35], respirology [12,36], thoracic surgery [12], gynecology [35], urology [37], and palliative medicine [38] were also included. All 9 studies demonstrated improvements in multidisciplinary oncology knowledge and skills postintervention.

**Table 2.** Summary of studies evaluating the impact of multidisciplinary educational interventions.^a^

Reference	Study design	Sample	Outcome measure	Main findings and conclusions
Cook et al[31]	Electronic surveys were sent to general surgery residents at the completion of 4-week rotations in MDB^b^, USOS^c^, and community-based TSR^d^ at Oregon Health and Science University in 2010‐2013. MDB included operative time, as well as half-days in pathology, radiology, medical oncology, and surgery clinic.	Total sample size: 32 in MDB, 73 in USOS, and 51 in TSR Operative logs of 29 residents in MDB, 11 in TSR, and 12 in USOS were obtained	Trainee satisfaction based on surveys Operative volume based on operative logs	MDB rotation residents rated the opportunity to perform and learn procedures higher than those in USOS (*P*=.02) and TSR (*P*=.01) 83% of MDB residents’ operative experience included breast cancer operations, compared to 71% of USOS and 12% of TSR groups MDB rotation residents rated higher on the quality of faculty teaching and educational materials than those on TSR (*P*=.03 and *P*=.04, respectively)
Khoshgoftar et al[37]	Short interviews were held with urology residents and faculty members regarding needs for holding web-based tumor boards prior to implementation of 20 monthly web-based tumor boards. Tumor boards were assessed through questionnaires postintervention, resident pretest and posttest scores for 5 consecutive tumor boards, and external evaluators from the faculty of urology.	35 urology residents 25 urology faculty members Panelists from pathology, radiation oncology, medical oncology, radiology, and nuclear medicine	Needs assessment, satisfaction levels, pretest and posttest scores, recommendations from external evaluators	Resident needs assessment was divided by level of importance and postgraduate years (ie, years 1‐2 vs 3‐4). An important limitation to participate was significant clinical responsibilities, particularly for lower year residents High resident satisfaction rate (71%‐88%) based on various aspects of web-based tumor boards. The most important technical issue was the low bandwidth speed. There was significant improvement in resident posttest scores in the majority of sessions
Mackay et al[36]	Respiratory and oncology trainees completed a 3-hour MDTM^e^ simulation session and completed pre- and postsimulation questionnaires	19 oncology and respiratory trainees (specialty training years 3‐7)	Perceptions of current training programs, confidence presenting in MDTMs, use of the simulation, and impact on future clinical practice	Trainees rated 4/10 for how well their program prepared them to present at MDTM Trainee confidence in presenting in MDTMs increased from 5/10 to 7/10 postintervention (*P*<.01) Trainees rated 9/10 for usefulness and 9/10 for likelihood the session will lead to changes in their practice
Martin et al[38]	Fellows completed three 1-hour lectures in palliative radiotherapy, as well as pre- and postcourse questionnaires and objective knowledge assessment multiple-choice questions.	5 hospice and palliative medicine fellows at the University of California, San Diego	Knowledge and confidence in palliative radiotherapy	Postintervention improvement in trainee-reported confidence in discussion with patients about radiotherapy (0.009), managing its common side effects (*P*=.021), and identifying oncologic emergencies related to radiotherapy (*P*=.012) Significant improvement in radiotherapy knowledge based on objective knowledge assessment questions (22% vs 86% pre- vs postintervention; *P*=.010) Increased trainee-reported likelihood of collaboration with radiation oncologists postintervention (*P*=.014)
Mattes et al[12]	Faculty, fellows, and residents attended a didactic lecture on radiation therapy in lung cancer care. Knowledge was tested using multiple choice questions pre- and postintervention.	A total of 121 faculty and trainees from pulmonology, thoracic surgery, and medical oncology Pretest: 54 residents/fellows and 9 faculty participated Posttest: 23 residents/fellows and 2 faculty participated	Knowledge of radiation therapy in lung cancer treatment and comfort in appropriate referral to radiation oncology	The majority had no didactic training (75%) or rotations (85.5%) in radiation oncology preintervention Significant improvements in mean objective test scores postintervention (*P*<.001) Postintervention, 100% of participants felt more knowledgeable in radiation therapy and 96% felt more comfortable making appropriate radiation oncology referrals
Meani et al[35]	Faculty and trainees completed a postintervention questionnaire following a multidisciplinary breast cancer course.	A total of 42 participants in medical oncology, radiation oncology, gynecology, and general surgery 11 heads of department/professors 17 consultants/attending Physicians 14 trainees: residents, medical fellows, PhD students, and postdoctoral fellows	Opinions on the impact of the course	Postintervention, 64% made changes in their clinical practice and 33% made institutional changes in breast cancer management 95% reported increased knowledge of MDB cancer care
Sloan et al[32]	Residents at the University of Kentucky received multidisciplinary instruction and completed 15 case-based stations about various domains of breast cancer care (ie, surgical oncology, medical oncology, radiology, radiation oncology, plastic surgery, and pathology). Surveys about the overall quality of intervention were completed by patients, faculty, and residents. Residents also completed pre- and postintervention surveys regarding specific breast cancer care-specific skills.	22 general surgery residents 3 radiation oncology residents 15 faculty at stations 12 patients with breast cancer at stations	Self-reported trainee improvement in breast cancer care–specific skills Perception of faculty, patients, and residents of the overall quality of intervention	Statistically significant trainee-reported improvement for all measured skills, including fine-needle aspiration, mammography interpretation, and treatment discussion with patients Overall, intervention rated favorably by trainees, faculty, and patients
Sloan et al[33]	Residents at the University of Kentucky completed 12 case-based stations during a head and neck oncology workshop, designed by faculty from general surgery, speech pathology, dentistry, radiation therapy, otolaryngology, plastic and reconstructive surgery, pathology, anesthesiology, and cardiothoracic surgery. Surveys about the overall quality of intervention were completed by patients, faculty, and residents. Residents also completed pre- and postintervention surveys regarding head and neck-specific skills.	21 general surgery residents 11 faculty at stations 8 standardized patients at stations (including 6 patients with cancer)	Self-reported trainee improvement in skills relevant to head and neck cancer care Perception of faculty, patients, and residents of the overall quality of intervention	Statistically significant trainee-reported improvement for most skills postintervention (*P*<.001) Overall, intervention rated favorably by trainees, faculty, and patients Residents generally endorsed having intervention minimum twice during residency
Sloan et al[34]	2 groups received multidisciplinary teaching in breast cancer care, including radiation oncology, radiology, surgery, and medical oncology, in the form of a 15-station workshop. The other 2 groups served as controls. 1 intervention and 1 control group were administered an 11-problem OSCE^f^ assessment immediately postintervention and the remaining 2 groups were administered the same OSCE assessment 8 months later. Residents were assessed by faculty and standardized patients during OSCE assessments.	48 general surgery residents from the University of Kentucky, divided evenly into 4 groups 15 faculty at stations 12 standardized patients at stations (including 5 patients with cancer)	Skills in diagnosis and management of breast cancer postintervention, assessed by faculty and standardized patients during OSCE assessments	Improvement in skills of residents who attended the workshop, compared to the control group, both immediately and 8 months postintervention (*P*<.01) Residents’ skills diminished after 8 months, as evidence by the difference in skill set between the group tested immediately versus the one tested 8 months postintervention (*P*<.004)

a Patients who performed assessments included actual and simulated patients.

b MDB: multidisciplinary breast.

c USOS: university surgical oncology service.

d TSR: traditional surgical rotation.

e MDTM: multidisciplinary team meeting.

f OSCE: Objective Structured Clinical Examination.

The study by Cook et al [31] compared the impact of a multidisciplinary breast rotation to traditional oncology or community rotations using trainee self-evaluations. Martin et al [38] and Mattes et al [12] analyzed the effectiveness of didactic learning for palliative radiotherapy and lung cancer radiotherapy, respectively, using pre- and postcourse trainee evaluations. Meani et al [35] studied the impact of a multidisciplinary breast cancer course on the knowledge and practice of faculty and trainees using a questionnaire. Three studies by Sloan et al tested the quality of case-based instruction, involving workshops or Objective Structured Clinical Examination (OSCE) stations, where evaluations were completed by trainees, standardized patients, and faculty [32-34]. In the 2004 study by Sloan et al [34], faculty and standardized patient completed evaluations following the observation of trainees in OSCE stations. Patient ratings mainly included interpersonal skills, while faculty ratings included both the clinical and interpersonal skills of trainees. In the other 2 Sloan et al studies, faculty and standardized patients provided feedback on the overall quality of workshops, rather than a specific focus on trainee skills [32,33]. Many of the standardized patients were actual patients with cancer [32-34]. Two of the Sloan et al studies with breast cancer-specific stations focused on knowledge and skills in the following fields: surgical, medical, and radiation oncology; pathology; plastic surgery; and radiology [32,34]. A pilot study by the same group included a head and neck workshop in which stations were designed by faculty from general surgery, radiation oncology, cardiothoracic surgery, otolaryngology, plastic surgery, pathology, anesthesiology, speech pathology, and dentistry [33].

In 8 of these 9 studies, the benefit of educational interventions was noted by the trainees through self-assessment of knowledge or skills [12,31-33,35-38], while Sloan et al [34] demonstrated improvements in knowledge or skills, as assessed by faculty and patients following the observation of trainees in OSCE stations. In addition to reporting subjective benefits, Khoshgoftar et al [37], Mattes et al [12], and Martin et al [38] used objective assessments to demonstrate improvements in trainee knowledge postintervention. Interestingly, Sloan et al [34] showed that while the intervention benefited residents’ knowledge and skill set in breast cancer management both immediately after and 8 months postintervention, it declined after 8 months. In the other 2 studies by this group [32,33], trainees, faculty, and patients rated the interventions highly.

### Quality Assessment

A summary of the MMAT quality assessment is included in Table S3 in Multimedia Appendix 1. Five studies were categorized as nonrandomized, 4 as qualitative, 13 as quantitative descriptive, 1 as mixed methods, and 1 as randomized controlled. Studies were given a score out of 5, based on the number of MMAT criteria met. Two studies were given an overall MMAT quality rating of 3 stars, 14 studies were rated as 4 stars, and the remaining 8 were rated as 5 stars. Overall, all studies were deemed to be satisfactory by authors, based on MMAT quality assessment criteria.

## Discussion

### Principal Results

To our knowledge, this is the first systematic review of multidisciplinary oncology education in postgraduate medical training. These data summarize educational gaps and potential solutions to improve multidisciplinary education for future trainees. Of the 24 studies included in the final analysis, 15 obtained faculties’ and trainees’ opinions on deficiencies and areas of improvement for existing multidisciplinary oncology education [16-19,24,26,27]. They generally reported limited multidisciplinary oncology training or knowledge, barriers to multidisciplinary training, and advocated for further instruction in different areas. The remaining 9 studies studied the impact of educational interventions on trainees’ oncology expertise [31-34,38]. Multidisciplinary rotations, tumor board meetings, didactic teaching, and case-based learning were found to be beneficial based on trainee self-assessments, written exams, and evaluations from faculty and patients following the observation of trainees in OSCE stations.

Filling the current gaps in multidisciplinary oncology education using the aforementioned educational interventions has the potential to improve multidisciplinary communication, appropriate referrals, and oncologic outcomes [3-5]. Studies by Mattes et al [12] and Martin et al [38] found that trainees were more likely to collaborate and make appropriate referrals to radiation oncologists after didactic teachings in lung cancer treatment and palliative radiotherapy, respectively. Several studies also found MTBMs to enhance trainee education [30,36,37]. In fact, the study by Mackay et al [36] found that tumor board simulation sessions significantly improved trainee’s confidence in presenting in tumor board sessions. After all, improved communication and referral patterns are central to effective multidisciplinary collaboration among oncology specialists and ultimately improve the access of patients to evidence-based oncologic treatments.

### Comparison With Prior Work

Geriatric oncology was consistently found to be an area in which trainees received limited training [16,26,27,39]. As cancer incidence increases in older adults, a population with a higher burden of comorbidities, trainees must gain sufficient knowledge and experience in geriatric oncology to optimize treatment [40]. These findings are echoed in a review by Morris et al [39] highlighting insufficient training and education in geriatric oncology among radiation oncology trainees across several different countries. This training should identify the specific needs of older patients and thereby result in a more informed and nuanced approach to this population’s medical and psychosocial issues [24]. Development of these skills may be achieved through dedicated rotations or training in geriatric oncology.

Based on findings from this study, it is evident that the quality of multidisciplinary oncology education and training needs to be assessed and addressed. Implementation of benchmarks to ensure sufficient training across residency and fellowship programs commonly involved in cancer care would provide an educational quality metric [6-10]. This would encourage training programs to develop and establish multidisciplinary oncology curricula. One approach to achieve this would be to ensure trainee participation in a variety of educational activities such as multidisciplinary case conferences, research, rotations, didactic teaching, and case-based learning led by faculty from other disciplines [11,31-34,38]. Furthermore, a review of each residency or fellowship program’s curriculum by a multidisciplinary faculty committee may ensure sufficient trainee exposure to collaborating oncology areas.

Competency-based medical education is an outcome-based approach to evaluate medical trainees and ensure a high degree of graduate skill set [41]. This is often done via objective measures, such as entrustable professional activities (EPAs) and milestones. The development of standardized and program-specific EPAs, specifically for multidisciplinary oncology education, would provide training programs with a specific measure of their trainees’ knowledge, skills, and progress in this area. Using EPAs would also identify areas of improvement for trainees early on in their training and would allow for additional support to improve multidisciplinary oncology competencies. Ultimately, these EPAs should mirror curriculum changes to ensure effective multidisciplinary oncology education. The benefits of using EPAs for geriatric oncology training are echoed by Eid et al [24]. They provide an example of an EPA to assess the appropriateness of chemotherapy for a geriatric patient, which includes the ability to perform a comprehensive geriatric assessment, having sufficient knowledge of chemotherapy toxicities and interactions, and assessment of suitability based on patients’ comorbidities. This represents a geriatric oncology-specific EPA for medical or hematology oncology trainees. Oncology training programs may adopt similar EPAs to ensure a high quality of multidisciplinary oncology training within their residency and fellowship programs.

Despite its merits, there are potential barriers to the implementation of oncology training curricula. Several factors may prevent trainee participation in multidisciplinary education activities, including limited elective time, educational options, or available personnel. For instance, those training in the community or rural hospitals may not have access to many electives in other oncology fields. For the same reason, there may be limited available multidisciplinary faculty to either design effective oncology curricula or mentor trainees. Furthermore, many residency or fellowship programs may have strict curricula and elective requirements, and thus limit elective options for trainees. To overcome some of these challenges, studies have suggested the importance of web-based courses or teaching sessions to supplement their curriculum. As a result of the COVID-19 pandemic, web-based education has become an integral part of medical training that will likely remain used to various degrees in the future [42,43]. Data supports the effectiveness of web-based training, including web-based rotations or clinical training [44-46], tumor board meetings [28,37], surgical skills training [47], and didactic and case-based teaching [48-52].

Furthermore, local, state-wide or provincial, and national resources and programs could also be offered to trainees interested in further advancing their multidisciplinary oncology knowledge and skills outside their residency and fellowship programs. Certainly, didactic teaching [12,35,38], as well as workshops and OSCE-style evaluation sessions [32-34] are valuable in advancing trainee education in multidisciplinary oncology care. Depending on the topic, these teaching sessions could be offered in person, remotely via web-based applications, or as a prerecording to enhance trainee participation. As indicated by Mackay et al [36], tumor board simulation sessions contribute to significant improvements in trainee confidence and skills in participating in tumor boards. This is a novel educational intervention not traditionally offered by residency or fellowship programs. The addition of such resources and programs outside of the mainstream postgraduate training programs has the potential to supplement trainee education toward multidisciplinary oncology care.

Given the time constraint of residency and fellowship, it is not feasible for trainees to gain all relevant multidisciplinary knowledge and skills while also excelling in all core competencies relevant to their program. Every proposed intervention will have its own challenges to implement and needs to be balanced against other rotations within the curriculum. Yet, it is preferred that trainees obtain sufficient multidisciplinary knowledge during training rather than through experience during practice. It is crucial that training programs conduct an evaluation of any new educational intervention and prioritize selected interventions in their curricula based on outcomes and feedback.

### Limitations

This study has limitations. Only 24 studies have analyzed the quality of multidisciplinary oncology education among postgraduate medical trainees. Furthermore, we limited our study to English-only and primary papers. It is possible that additional studies analyzing multidisciplinary oncology education in other languages or papers (eg, grey literature) exist that are missing from our results. Over a third of these studies were also published more than 5 years ago. Particularly, 3 of the intervention studies are by Sloan et al [32-34], published in 1997, 1999, and 2004, which could have had overlapping participants. This could limit the generalizability of the findings from these studies. There is a need for additional and more contemporary research assessing the needs of postgraduate medical trainees and the impact of newer educational interventions. It is particularly important to evaluate the use of technologies currently used in medical education such as web-based live teaching [43-47], clinical teaching tools such as case-based modules with built-in radiology software [53,54], and virtual reality surgical training [55-57]. Additionally, none of the studies on educational interventions were conducted with trainees in geriatric oncology. As previously discussed, this is an important aspect of oncology, though generally missing from oncology training curriculums. Thus, additional studies are needed within these fields. Furthermore, while a large proportion of studies solely focus on gaps in geriatric oncology education, this may not be generalizable to all multidisciplinary oncology education needs. Future research will be important in developing multidisciplinary oncology curricula for postgraduate trainees.

### Conclusions

This systematic review demonstrated several gaps in the existing multidisciplinary oncology training of postgraduate medical trainees and the promising results of various educational interventions in bridging these gaps. Further studies investigating the needs of trainees at both local and national levels are needed to develop specific educational curricula and program requirements that focus on multidisciplinary oncology collaboration. Future research should also assess contemporary educational interventions to determine the most effective methods of attaining multidisciplinary oncology expertise among postgraduate medical trainees.

## Supplementary material

10.2196/63655 Multimedia Appendix 1 Research data, search strategy, and assessment results.

10.2196/63655 Checklist 1 PRISMA (Preferred Reporting Items for Systematic Reviews and Meta-Analyses) checklist.

## Abbreviations:

EPA: entrustable professional activity
MMAT: Mixed Methods Appraisal Tool
MTBM: multidisciplinary tumor board meeting
OSCE: Objective Structured Clinical Examination
PRISMA: Preferred Reporting Items for Systematic Reviews and Meta-Analyses
