# Supplementary material for: Multidisciplinary Oncology Education Among Postgraduate Trainees: Systematic Review
Source: JMIR Med Educ. 2025 May 26;11:e63655. doi: 10.2196/63655 (PMC12129420; doi:10.2196/63655)
Supplement: Multimedia Appendix 1 [file mededu-v11-e63655-s001.docx]

**Table S1**. Number of articles identified per database after the initial search on July 21, 2021, and the updated search on February 26, 2023 and October 29, 2024. Numbers respond to the articles after the removal of duplicates within each database.

| **Databases** | **July 21, 2021** | **Feb 26, 2023** | **Oct 9, 2024** | **Totals** |
| --- | --- | --- | --- | --- |
|  |  |  |  |  |
| MEDLINE (Ovid) | 1248 | 208 | 214 | **1670** |
| MEDLINE ePub Ahead of Print/MEDLINE In-Process & Other Non-Indexed Citations (Ovid) | 230 | 92 | 88 | **410** |
| Embase (Ovid) | 3098 | 628 | 695 | **4421** |
| Cochrane Central Register of Controlled Trials (Ovid) | 53 | 8 | 2 | **63** |
| Cochrane Database of Systematic Reviews (Ovid) | 1 | 0 | 0 | **1** |
| APA PsycINFO (Ovid) | 310 | 40 | 49 | **399** |
| ERIC (EbscoHost) | 19 | 3 | 5 | **27** |
| **Totals** | **4959** | **979** | **1053** | **6991** |

**Table S2.** Search strategy for Ovid MEDLINE(R) from the update search, October 9, 2024, is included. Numbers correspond to the total number of articles published between 1946 and October 8, 2024.

| **Number** | **Searches** | **Results** |
| --- | --- | --- |
| 1 | "Facility Regulation And Control"/ | 3195 |
| 2 | "Joint Commission On Accreditation Of Healthcare Organizations"/ | 7478 |
| 3 | Clinical Competence/ | 109613 |
| 4 | Ed.fs. [Education Floating Subheading] | 309497 |
| 5 | Education, Medical, Continuing/ | 25704 |
| 6 | exp Academic Medical Centers/ | 104377 |
| 7 | exp Accreditation/ | 20337 |
| 8 | exp Certification/ | 19789 |
| 9 | exp Competency-Based Education/ | 4865 |
| 10 | exp Consensus Development Conference/ | 12846 |
| 11 | exp Consensus Development Conferences As Topic/ | 3012 |
| 12 | exp Consensus/ | 23810 |
| 13 | exp Credentialing/ | 58711 |
| 14 | exp Curriculum/ | 103200 |
| 15 | exp Education, Medical, Graduate/ | 85622 |
| 16 | exp Education, Medical/ | 191125 |
| 17 | exp Education, Professional/ | 341161 |
| 18 | exp Educational Measurement/ | 171765 |
| 19 | exp Faculty, Medical/ | 15333 |
| 20 | exp Faculty/ | 41258 |
| 21 | "Fellowships and Scholarships"/ | 10233 |
| 22 | exp Hospitals, Teaching/ | 56821 |
| 23 | exp Inservice Training/ | 30212 |
| 24 | exp International Educational Exchange/ | 3591 |
| 25 | Interprofessional Education/ [ new MeSH 2021 ] | 657 |
| 26 | exp Licensure/ | 18494 |
| 27 | exp Mentors/ | 13820 |
| 28 | exp Models, Educational/ | 10620 |
| 29 | exp Preceptorship/ | 5783 |
| 30 | exp Professional Competence/ | 134021 |
| 31 | exp Schools, Medical/ | 28577 |
| 32 | exp Teaching Materials/ | 125371 |
| 33 | exp Teaching/ | 95524 |
| 34 | Hospitals, Teaching/ | 25333 |
| 35 | Hospitals, University/ | 32857 |
| 36 | Inservice Training/ | 20800 |
| 37 | Pilot Projects/ | 155402 |
| 38 | Professional Competence/ | 25296 |
| 39 | Program Development/ | 30507 |
| 40 | Program Evaluation/ | 68218 |
| 41 | Schools, Medical/ | 28577 |
| 42 | Specialty Boards/ | 3959 |
| 43 | st.fs. [Standards] | 777713 |
| 44 | Training Support/ | 6349 |
| 45 | (fellow or fellows or fellowship*).mp,kw. | 35080 |
| 46 | (skill or skilled or skills or skilling or skillful*).mp,kw. | 256119 |
| 47 | (training? or trainee?).mp,kw. | 542654 |
| 48 | accredit*.mp,kw. | 36980 |
| 49 | academ*.mp,kw. | 230869 |
| 50 | certif*.mp,kw. | 70928 |
| 51 | (clinical* adj2 faculty).mp. | 1522 |
| 52 | competen*.mp,kw. | 261888 |
| 53 | credential*.mp,kw. | 8091 |
| 54 | curricul*.mp,kw. | 117370 |
| 55 | curricula?.mp,kw. | 24208 |
| 56 | educat*.mp,kw. | 1155269 |
| 57 | governance.mp,kw. | 18580 |
| 58 | (health* adj2 faculty).mp. | 1163 |
| 59 | instruction.mp,kw. | 40163 |
| 60 | learn*.mp,kw. | 612357 |
| 61 | licenc*.mp,kw. | 3026 |
| 62 | licens*.mp,kw. | 55641 |
| 63 | (medical* adj2 faculty).mp. | 18560 |
| 64 | (medical adj2 school?).mp. | 54225 |
| 65 | mentor*.mp,kw. | 27865 |
| 66 | outline?.mp,kw. | 129523 |
| 67 | postgrad*.mp,kw. | 19550 |
| 68 | post-grad*.mp,kw. | 3622 |
| 69 | preceptor*.mp,kw. | 7957 |
| 70 | proctor*.mp,kw. | 1114 |
| 71 | (program? or programme?).mp,kw. | 1066994 |
| 72 | qualif*.mp,kw. | 57982 |
| 73 | seminar?.mp,kw. | 9699 |
| 74 | standard?.mp,kw. | 1800792 |
| 75 | syllab*.mp,kw. | 8717 |
| 76 | teach*.mp. | 246838 |
| 77 | train*.mp,kw. | 678409 |
| 78 | upskill*.mp,kw. | 508 |
| 79 | up-skill*.mp,kw. | 121 |
| 80 | workshop*.mp,kw. | 42566 |
| 81 | or/1-80 [ Education & Related Terms ] | 5045412 |
| 82 | exp Patient Care Team/ | 74196 |
| 83 | exp Interprofessional Relations/ | 73317 |
| 84 | exp Interdisciplinary Communication/ | 18308 |
| 85 | Interdisciplinary Placement/ [New MeSH as of 2018] | 325 |
| 86 | Interdisciplinary Research/ [New MeSH as of 2018] | 684 |
| 87 | Interdisciplinary Studies/ | 1346 |
| 88 | Cooperative Behavior/ | 46986 |
| 89 | Physician-Nurse Relations/ | 2421 |
| 90 | cross-disciplin*.mp. | 2183 |
| 91 | cross train*.mp. | 604 |
| 92 | crosstrain*.mp. | 12 |
| 93 | inter-professional*.mp. | 1654 |
| 94 | interprofessional*.mp. | 62326 |
| 95 | inter-disciplinar*.mp. | 794 |
| 96 | interdisciplinar*.mp. | 61589 |
| 97 | interoccupation*.mp. | 2 |
| 98 | inter-occupation*.mp. | 10 |
| 99 | (integrat* adj2 care).mp. | 26062 |
| 100 | (integrat* adj2 education*).mp. | 1943 |
| 101 | (integrat* adj2 health*).mp. | 27561 |
| 102 | (integrat* adj2 team?).mp. | 1077 |
| 103 | medical etiquette.mp. | 23 |
| 104 | multioccupation*.mp. | 7 |
| 105 | multi-occupation*.mp. | 26 |
| 106 | multi-professional*.mp. | 1575 |
| 107 | multiprofessional*.mp. | 1757 |
| 108 | multi-disciplinar*.mp. | 7810 |
| 109 | multidisciplinar*.mp. | 105681 |
| 110 | nurse physician relation*.mp. | 218 |
| 111 | physician nurse relation*.mp. | 2472 |
| 112 | professional etiquette.mp. | 25 |
| 113 | trans-disciplinar*.mp. | 240 |
| 114 | transdisciplinar*.mp. | 2495 |
| 115 | inter-department*.mp. | 167 |
| 116 | interdepartment*.mp. | 2011 |
| 117 | intra-hospital*.mp. | 782 |
| 118 | intrahospital*.mp. | 1499 |
| 119 | (collaborat* adj2 care).mp. | 5331 |
| 120 | (collaborat* adj3 department*).mp. | 1000 |
| 121 | (collaborat* adj3 knowledge).mp. | 908 |
| 122 | (collaborat* adj3 service?).mp. | 1867 |
| 123 | (collaborat* adj3 skill*).mp. | 864 |
| 124 | (collaborat* adj3 specialt*).mp. | 346 |
| 125 | cooperative behavio?r*.mp. | 47892 |
| 126 | co-operative behavio?r*.mp. | 55 |
| 127 | (multimodal adj2 care).mp. | 344 |
| 128 | (multi-modal adj2 care).mp. | 28 |
| 129 | (modular* adj2 team*).mp. | 12 |
| 130 | (team or teams or teamwork* or team-work*).mp. [very broad search] | 247418 |
| 131 | (multimodal adj2 manag*).mp. | 1137 |
| 132 | (multi-modal adj2 manag*).mp. | 64 |
| 133 | (modular* adj2 care).mp. | 25 |
| 134 | (modular* adj2 manag*).mp. | 26 |
| 135 | (Mayo adj3 model).mp. | 195 |
| 136 | or/82-135 [ Interdisciplinary or Multidisplinary or Transdisciplinary or Interprofessional] | 481839 |
| 137 | 81 and 136 [ Education + Interdisciplinary ] | 256454 |
| 138 | exp Neoplasms/ | 4027900 |
| 139 | exp Antineoplastic Agents/ | 1292448 |
| 140 | Cancer Care Facilities/ | 6049 |
| 141 | Cancer Pain/ | 2708 |
| 142 | Cancer Survivors/ | 10662 |
| 143 | Cancer Vaccines/ | 15979 |
| 144 | exp "Hospice and Palliative Care Nursing"/ | 2585 |
| 145 | exp Hospice Care/ | 8420 |
| 146 | exp Oncology Service, Hospital/ | 1525 |
| 147 | exp Palliative Care/ | 65838 |
| 148 | exp Terminal Care/ | 58790 |
| 149 | Integrative Oncology/ | 191 |
| 150 | Medical Oncology/ | 25266 |
| 151 | exp Oncologists/ | 2232 |
| 152 | Oncology Nursing/ | 8506 |
| 153 | Psycho-Oncology/ | 272 |
| 154 | Radiation Oncology/ | 6177 |
| 155 | Surgical Oncology/ | 904 |
| 156 | antineoplas*.mp. | 598379 |
| 157 | anti-neoplas*.mp. | 2997 |
| 158 | "end of life care".mp. | 13233 |
| 159 | cancer*.mp. | 2076100 |
| 160 | carcino*.mp. | 1128809 |
| 161 | hospice?.mp. | 20925 |
| 162 | malignancies.mp. | 132833 |
| 163 | malignancy.mp. | 156154 |
| 164 | malignant.mp. | 407168 |
| 165 | metasta*.mp. | 619143 |
| 166 | necrosectom*.mp. | 1078 |
| 167 | neoplas*.mp. | 3478543 |
| 168 | onco*.mp. | 628573 |
| 169 | palliative care.mp. | 76674 |
| 170 | palliative nursing.mp. | 289 |
| 171 | palliative supportive care.mp. | 82 |
| 172 | palliative surger*.mp. | 2188 |
| 173 | palliative therap*.mp. | 2305 |
| 174 | palliative treatment?.mp. | 7438 |
| 175 | psychooncolog*.mp. | 187 |
| 176 | psycho-oncolog*.mp. | 2134 |
| 177 | psychosocial oncolog*.mp. | 377 |
| 178 | psycho-social oncolog*.mp. | 6 |
| 179 | terminal care.mp. | 33859 |
| 180 | therapeutic radiolo*.mp. | 708 |
| 181 | tumo?r*.mp. | 2350881 |
| 182 | or/138-181 [ Cancer & related terms ] | 5775042 |
| 183 | 137 and 182 [ Education + Interdisciplinary + Cancer ] | 31724 |
| 184 | "Fellowships and Scholarships"/ | 10233 |
| 185 | exp "Internship and Residency"/ | 63579 |
| 186 | fellow?.mp. | 21021 |
| 187 | fellowship?.mp. | 17886 |
| 188 | graduate.mp. | 71694 |
| 189 | graduates.mp. | 20807 |
| 190 | house staff.mp. | 1287 |
| 191 | internship?.mp. | 67047 |
| 192 | intern?.mp. | 16970 |
| 193 | medical residencies.mp. | 49 |
| 194 | medical residency.mp. | 555 |
| 195 | medical resident?.mp. | 1877 |
| 196 | PGY.mp. | 1783 |
| 197 | PGYs.mp. | 69 |
| 198 | postgrad*.mp. | 19550 |
| 199 | post-grad*.mp. | 3622 |
| 200 | surgery residen*.mp. | 4424 |
| 201 | surgical residen*.mp. | 3818 |
| 202 | trainee?.mp. | 32356 |
| 203 | oncology residen*.mp. | 311 |
| 204 | radiation residen*.mp. | 6 |
| 205 | resident physician*.mp. | 2864 |
| 206 | house physician?.mp. | 96 |
| 207 | resident?.mp. | 186828 |
| 208 | medical training.mp. | 5765 |
| 209 | or/184-208 [ Residents / Fellows / Trainees ] | 352671 |
| 210 | 183 and 209 [ Education + Interdisciplinary + Cancer + Fellows/Trainees ] | 1828 |
| 211 | limit 210 to english language | 1670 |

**Table S3.** Summary of the Mixed Methods Appraisal Tool (MMAT) [15] quality assessment results. Specific criteria are indicated here: [15]. Note: stars indicate the number of criteria met (out of 5). Cr. 1-5 refer to criterion 1-5.

| **First Author (Year)** | **Study Type** | **Cr. 1** | **Cr. 2** | **Cr. 3** | **Cr. 4** | **Cr. 5** | **Quality Rating** | **Comments** |
| --- | --- | --- | --- | --- | --- | --- | --- | --- |
| **Akthar (2018)**  [17] | Quantitative descriptive | Yes | Yes | Yes | No | Yes | **** | Response rate = 34% |
| **Brenner (2020)**  [18] | Quantitative descriptive | Yes | Yes | Yes | No | Yes | **** | Response rate = 36% |
| **Cook (2016)**  [32] | Non-randomized | Yes | Yes | No | Yes | Yes | **** |  |
| **David (2022)** [26] | Quantitative descriptive | Yes | Yes | Yes | No | Yes | **** | Response rate = 41% |
| **Delaye (2023)**  [23] | Quantitative descriptive | yes | yes | yes | can't tell | Yes | **** |  |
| **Eid (2015)**  [25] | Qualitative | Yes | Yes | Can't tell | No | Yes | *** | No information on how interviews or rotation evaluations were analyzed. No quotes available. |
| **Givi (2022)** [30] | Qualitative | Yes | Yes | Yes | Yes | Yes | ***** |  |
| **Khoshgoftar (2023)**  [38] | Mixed methods | Yes | yes | yes | yes | Can't tell | **** | Limited detail on the questionnaires and no quotes provided for needs assessment interviews |
| **La Nail (2023)**  [31] | Quantitative descriptive | yes | yes | yes | yes | Yes | ***** |  |
| **Mackay (2024)**  [37] | Quantitative descriptive | Yes | Yes | Yes | Can't tell | Yes | **** |  |
| **Maggiore (2018)**  [27] | Quantitative descriptive | Yes | Yes | Yes | No | Yes | **** | Response rate = 48% |
| **Martin (2019)**  [39] | Non-randomized | Yes | Yes | Yes | Yes | Yes | ***** |  |
| **Mattes (2023)** [12] | Non-randomized | Yes | Yes | No | Yes | Yes | **** | Missing outcome data. Sample size of 76 pre-test and 25 post-test |
| **Maurer (2023)**  [24] | Quantitative descriptive | Yes | Yes | Yes | Can't tell | Yes | **** |  |
| **Meani (2022)** [36] | Quantitative descriptive | Yes | Yes | Yes | Yes | Yes | ***** | Response rate = 100% |
| **Morris (2017)**  [28] | Quantitative descriptive | Yes | Yes | Yes | No | Yes | **** | Response rate = 52% |
| **Morris (2022)** [21] | Quantitative descriptive | Yes | Yes | Yes | No | Yes | **** | Response rate = 58% and 52% (round 1 and 2) |
| **Park (2020)**  [19] | Quantitative descriptive | Yes | Yes | Yes | No | Yes | **** |  |
| **Picca (2023)**  [29] | Qualitative | Yes | Yes | Yes | Yes | Yes | ***** |  |
| **Sloan (1997)**  [33] | Non-randomized | Yes | Yes | Yes | Yes | Yes | ***** |  |
| **Sloan (1999)**  [34] | Non-randomized | Yes | Yes | No | Yes | Yes | **** |  |
| **Sloan (2004)**  [35] | Randomized controlled | Can't tell | Yes | No | Yes | Yes | *** | Missing outcome data due to scheduling issues |
| **Walraven (2022)** [22] | Qualitative | Yes | Yes | Yes | Yes | Yes | ***** |  |
| **Wilson (2012)**  [20] | Quantitative descriptive | Yes | Yes | Yes | Yes | Yes | ***** | Response rate = 83% |
